# The association between the GNB3 rs5443 C/C genotype and obesity phenotypes in Taiwanese individuals

**DOI:** 10.37796/2211-8039.1697

**Published:** 2026-03-01

**Authors:** Ling-Yi Xiao, Zi-Lun Lai, Yang-Di Su, Szu-Yun Wang, Nia-Jia Zheng, Po-Ren Hsueh

**Affiliations:** a Department of Laboratory Medicine, China Medical University Hospital, China Medical University, Taichung 40447, Taiwan; b Division of Infectious Diseases, Department of Internal Medicine, China Medical University Hospital, China Medical University, Taichung 40447, Taiwan

**Keywords:** GNB3, Obesity, Single nucleotide polymorphism, Taiwanese

## Abstract

**Background:**

The prevalence of obesity has increased significantly over the years, and its health concerns cannot be underestimated. Obesity not only causes potential mobility limitations in daily life but also increases the risk of developing cardiovascular diseases, diabetes, cancer and other health conditions. While an imbalanced diet and lack of exercise are well-known causes of obesity, genetic patterns also influence its development. Although the GNB3 gene is known to be involved in lipid metabolism and fat cell differentiation, studies have shown inconsistent associations between a common single nucleotide polymorphism of GNB3 (c.825C > T, rs5443) and obesity across different populations. Therefore, this study aims to analyze the association between the GNB3 c.825C > T polymorphism and obesity in the Taiwanese population using various grouping criteria.

**Subjects/Methods:**

The study recruited 372 eligible subjects for GNB3 SNP rs5443 (c.825C > T) testing at China Medical University Hospital in Taichung, Taiwan. Clinical parameters, including age, sex, weight, BMI and body fat percentage were assessed for all participants. The GNB3 rs5443 C/T genotypes were determined using two differentially labeled allele-specific probes and a specific paired PCR primer set.

**Results:**

Our results demonstrated that the distribution of GNB3 rs5443 genotypes (C/C, C/T, T/T) was not significantly correlated with sex and age ( *p* > 0.05). However, the distribution of GNB3 genotypes (C/C, T/T) showed a statistical significance between subjects with BMI < 24 and BMI ≥ 27. Furthermore, our results revealed that the C/T and T/T genotypes had higher frequency distributions compared to the C/C genotype in females with body fat percentages below 30 % ( *p* = 0.022 and *p* = 0.004, respectively).

**Conclusions:**

Taiwanese individuals carrying the C/C homozygous genotype of the GNB3 gene may have a higher susceptibility to obesity, particularly among females. This finding could potentially be combined with polymorphisms of other obesity-related genes to develop a clinical screening tool for assessing obesity risk.

## 1. Introduction

According to the 2024 WHO World Health Statistics, the global obesity population aged 18 and above increased by nearly 700 million between 1990 and 2022 [1]. In Taiwan, the 2021 National Health Interview Survey conducted by the Health Promotion Administration, Ministry of Health and Welfare, showed that 24.7 % of adults were overweight and 23.2 % were obese [2]. Overweight and obesity are established metabolic risk factors for noncommunicable diseases (NCDs), increasing the risk of cardiovascular disease, hypertension, hyperlipidemia, type 2 diabetes, cancer, chronic respiratory disease, and gastrointestinal disorders [3]. Obesity typically results from an imbalance between daily dietary intake and physical activity, leading to excessive fat accumulation in adipose tissue [4]. In addition to unhealthy lifestyle habits, genetic factors are known to influence obesity susceptibility, with heritability estimated at 61–80 % [5]. Scientists have extensively studied obesity-related genes, and numerous genetic polymorphisms have been linked to body mass index (BMI), cholesterol levels, waist circumference, and blood pressure [6–9]. Understanding the phenotypes of obesity-related genes through genetic analysis can help assess obesity risk, enabling early modifications to individual dietary and exercise plans, personalized treatment strategies, and prevention of NCDs.

The guanine nucleotide-binding protein subunit β3 (GNB3) gene encodes the beta subunit of heterotrimeric G-protein, which is an important regulator of the alpha subunit and GTPase activity, as well as various signal transduction receptors and effectors [10]. GNB3 is predicted to participate in several processes, including cell volume homeostasis, regulation of fat cell differentiation, and lipid metabolism [11]. A common single nucleotide polymorphism (c.825C > T, rs5443) on exon 10 of the GNB3 gene has been associated with hypertension, obesity, and atherosclerosis [12–15]. Studies have shown that the T allele of the GNB3 C825T polymorphism enhances G-protein activation, resulting in increased cell proliferation [16]. However, evidence linking the GNB3 C825T polymorphism to obesity has been contradictory. While studies have demonstrated that the T allele predisposes individuals to obesity in German, Chinese and Korean populations [17–20], no such association was found in Caucasian and white Danish populations [21,22]. Even within the Japanese population, findings regarding the relationship between the GNB3 C825T polymorphism and obesity have been inconsistent [23,24]. Therefore, further investigation of the association between GNB3 C825T polymorphism and obesity remains crucial for obesity risk assessment.

In Taiwan, while the GNB3 C825T polymorphism allele frequency distribution showed no statistical significance with obesity, the GNB3 CC genotype was found to be predictive of total cholesterol levels in non-obese individuals [10]. Previous studies in Japan and Taiwan have yielded inconsistent results due to different grouping criteria. Thus, in this study, we analyzed the distribution of GNB3 C825T polymorphism genotypes among Taiwanese subjects under different grouping criteria to identify statistically significant clinical implications. Our findings revealed that the GNB3 C825T genotype in Taiwanese individuals positively correlates with BMI and body fat percentage. The C/C homozygous genotype of GNB3 may serve as a predictive risk indicator for future BMI and body fat percentage, particularly among females.

## 2. Methods

### 2.1. Study population

This enrollment of participants in this study was conducted from December 4 to December 7 in 2023 during the 2023 Health Expo, in Taipei, Taiwan. Clinical information of subjects, such as weight, height, age, sex, BMI and body fat percentage was collected. Data on subjects’ sex, age, weight, and height were collected via questionnaire. The BMI was calculated using the formula “Weight (kg)/Height (m)^2^”. Since body composition differs between males and females, separate formulas were used to calculate body fat percentage. The formula (1.2 × BMI) + (0.23 × Age) − (10.8 × 1) − 5.4 was for males and (1.2×BMI)+(0.23×Age) − (10.8×0) − 5.4 was for females [25]. This study was approved by the Institutional Review Board of China Medical University Hospital (CMUH114-REC1-043).

### 2.2. Genotype determination

GNB3 genotype testing of these participants was performed at China Medical University Hospital, Taichung, Taiwan. The DNA from buccal samples was extracted using the QIAamp DNA MicroKit (QIAGEN, German) according to manufacturer’s instructions. GNB3 rs5443 genotyping was performed using the TaqMan SNP genotyping assay (Thermo-Fisher, USA) and QuantiTect Multiplex PCR Kit (QIAGEN, German). The probes for GNB3 C or T allele were labeled with VIC™ reporter dye and FAM™ reporter dye, allowing the distinction between GNB3 homozygous genotypes (C/C, T/T) and heterozygous genotype (C/T). Fluorescence signal detection was performed using the Applied Biosystems StepOnePlus system (ThermoFisher, USA).

### 2.3. Statistical analysis

All statistical analyses were performed using JASP software (version 0.19.3). Chi-square tests were used to compare genotype frequencies between subgroups. Results with *p*-values less than 0.05 were considered statistically significant.

## 3. Results

### 3.1. Study population

**Fig. 1:**
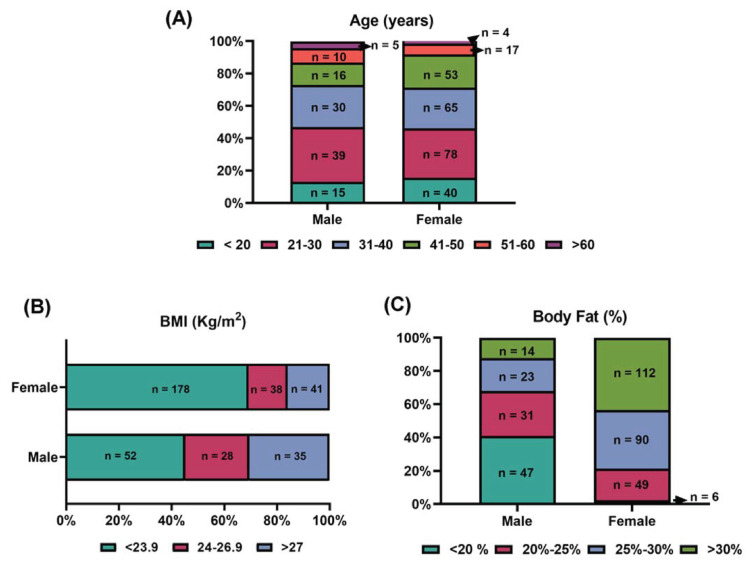
Distribution of (A) age (B) body mass index (BMI) and (C) body fat percentage of 372 subjects in this study. The Y-axis in (A) and (C) represents the percentage of subjects at different ages (years) and different body fat percentages (%), respectively. The X-axis in (B) represents the percentage of subjects at different BMI values (Kg/m^2^).

During the analysis process, 5 subjects had missing data in the age field, 1 subject had missing data in the sex field, and 2 subjects each had missing data in the height and weight fields. Therefore, a total of 372 eligible subjects were included in the complete analysis of GNB3 genotype with a mean age of 34 years. In addition, the average BMI was 23.6 kg/m^2^ and the average body fat percentage was 27.3 %. The distributions of age, BMI, and body fat percentage across male (n = 115) and female (n = 257) populations are presented in Fig. 1. There was no significant difference in age distribution between males and females (Fig. 1A). However, a higher percentage of females had a BMI < 23.9 kg/m^2^, accounting for 69.2 % (n = 178) of all females (Fig. 1B). Interestingly, in terms of body fat percentage distribution, most females (n = 112, 43.6 %) had a body fat percentage of >30 %. In contrast, a larger proportion of males had body fat below 20 % (n = 47, 40.9 %) (Fig. 1C). Therefore, when evaluating obesity, we should not only rely on a single reference criterion.

### 3.2. Association between GNB3 rs5443 genotypes and sex in Taiwanese individuals

We first analyzed the potential association between GNB3 genotypes (C/C, C/T, T/T) and sex using the collected data. Among all male subjects (n = 115), 23.5 % had the GNB3 C/C genotype (n = 27), 47 % had the C/T genotype (n = 54), and 29 % had the T/T genotype (n = 34). In the female subjects (n = 257), the percentages with the C/C, C/T, and T/T genotypes were 16.7 % (n = 43), 53.7 % (n = 138), and 29.6 % (n = 76), respectively. The statistical results are shown in Table 1. No statistically significant differences were observed in the distribution of the C/C genotype compared to the C/T or T/T genotypes in relation to sex ( *p* = 0.105 and *p* = 0.290, respectively). These findings suggest that the GNB3 rs5443 SNP genotypes are not significantly associated with sex in the Taiwanese population.

### 3.3. Association between GNB3 rs5443 genotypes and age in Taiwanese individuals

It is well established that the metabolic rate decreases with age, which affects fat accumulation. Since the GNB3 C825T polymorphism has been associated with metabolism, we stratified our study population into two age groups (≥40 years and <40 years) to analyze potential differences in the distribution of the GNB3 C/C, C/T, and T/T genotypes. As shown in Table 1, there was no statistically significant difference in the distribution of genotypes between subjects aged above and below 40 years ( *p* = 0.831). Similarly, no significant differences were observed when analyzing the C/C and T/T genotypes specifically ( *p* = 0.611). These findings suggest that age does not influence the frequency of the GNB3 C825T polymorphism in the Taiwanese population.

### 3.4. The GNB3 C/C genotype is associated with increased BMI and obesity

The study population was categorized into three groups based on BMI values: normal (BMI < 24), overweight (24 ≤ BMI < 27), and obesity (BMI ≥ 27). We analyzed the distribution of C/C genotype compared to C/T or T/T genotypes in relation to BMI values. The results showed no significant association between the C/C and the C/T genotype distribution ratios and BMI levels ( *p* = 0.177). However, when analyzing the C/C versus T/T genotype distribution among subjects with normal BMI (<24), the T/T genotype was notably more prevalent at 68.7 % (n = 77) compared to the C/C genotype at 31.3 % (n = 36). The association analysis between these distribution ratios and overweight/obesity status yielded a p-value of 0.020, indicating a statistically significant association between the GNB3 C/C and T/T genotype distributions and BMI levels (Table 2). To further examine the association between GNB3 rs5443 SNP and BMI values, we conducted pairwise comparisons of C/C and T/T distribution ratios among normal, overweight, and obesity groups. The results revealed a significant difference in C/C versus T/T distribution ratios only between the normal and obesity groups ( *p* = 0.01). These findings suggest that Taiwanese individuals carrying the GNB3 rs5443 SNP C/C genotype may have a higher risk of obesity (BMI ≥ 27).

### 3.5. GNB3 rs5443 genotypes are correlated with body fat percentage in females

**Fig. 2:**
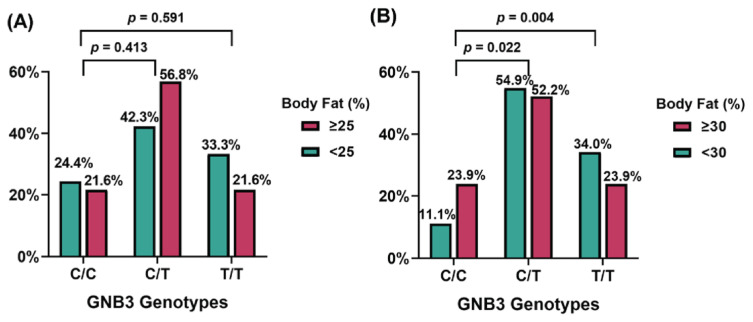
Genotype distribution and allele frequencies grouped by body fat percentage among (A) male (n = 115) and (B) female (n = 257) subjects. The Y-axis represents the percentage of subjects with different GNB3 genotypes.

According to Table 2, there was a statistically significant association between GNB3 genotypes and BMI. However, BMI is no longer the sole criterion for evaluating overweight or obesity status. Compared to BMI, body fat percentage provides a more direct measure of body fat content, which is directly linked to various health conditions such as cardiovascular diseases, stroke, and hypertension. Consequently, body fat percentage has gained greater attention in recent years. Since the definitions of high and normal body fat percentages differ between males and females, we first stratified the subjects by sex. Based on physiological differences, the thresholds for normal body fat percentage were set at 25 % for men and 30 % for women. The distribution of GNB3 genotypes (C/C, C/T, T/T) showed no statistically significant association with body fat levels in males ( *p* = 0.413 and *p* = 0.591) (Fig. 2A). Interestingly, among female subjects, a higher proportion of individuals with body fat percentage below 30 % was observed in those with the C/T (54.9 %, n = 79) and T/T (34.2 %, n = 49) genotypes compared to those with the C/C genotype (11.1 %, n = 16) (Fig. 2B). In fact, the distribution of genotypes across subjects with the different body fat percentage showed statistically significant associations ( *p* = 0.015) (data not shown). To further examine the association between GNB3 rs5443 SNP and body fat percentage, we conducted pairwise comparisons of C/C, C/T and T/T distribution ratios between females with <30 % and ≥30 % body fat. The results revealed a significant difference between the <30 % and ≥30 % groups only for the C/C and T/T genotypes ( *p* = 0.004). Therefore, these findings suggest that body fat percentage in females is more influenced by the GNB3 SNP genotypes, with females carrying the C/C genotype being more likely to exceed 30 % body fat.

## 4. Discussion

As societal advances and developments, obesity has gradually become a significant health concern that demands attention. Through the development of human genome decoding and gene sequencing technology, scientists have discovered several genes associated with obesity. Among these is the GNB3 gene, particularly its SNP (C825T) located in exon 10, which is considered to be positively correlated with obesity. However, research findings regarding the C or T allele at this locus show inconsistent and contradictory results, especially when different research teams use varying definitions for their subjects. In this study, we investigated the potential association between the distribution of GNB3 genotypes (C/C, C/T, T/T) and subjects classified under different conditions. Our results showed that neither sex nor age affects the distribution of GNB3 C or T alleles in the Taiwanese population. However, we found statistically significant differences in the distribution of GNB3 C/C and T/T genotypes between groups with BMI < 24 and BMI ≥ 27. Additionally, statistical significance was observed in the distribution of GNB3 C/C versus C/T or T/T genotypes in females with respect to body fat percentage. In conclusion, our findings suggest that in the Taiwanese population, the homozygous C/C genotype may be associated with an increased risk of higher BMI or body fat percentage.

Similar to other diseases, obesity or being underweight is not determined by a single gene. Genome-wide association studies (GWAS) have identified at least 58 genetic loci associated with obesity-related traits (including BMI, waist circumference, and body fat) [6]. However, since GWAS analyses have primarily been conducted on European and East Asian populations [7], there might be discrepancies when applied to the Taiwanese population due to racial differences, lifestyle, or dietary habits. Studies of obesity-related SNPs in the Taiwanese population have identified seven key genes: PPARγ, PPARγ2, GNB3, SDC3, ADRB2, FTO, and ESR1. Notably, research has shown that when SDC3 SNP (rs2282440) and PPARγ2 (rs1801282) occur simultaneously, they demonstrate a strong positive association with obesity [8]. While one study found no association between GNB3 SNP (rs5443) and obesity ( *p* > 0.05), it used a BMI threshold of 27 as its grouping criterion which differs from our study’s BMI classification. Additionally, our study analyzed the association between body fat percentage and GNB3 SNP (rs5443). Future research examining the relationships between multiple obesity-related genes using our study’s criteria (BMI and body fat percentage) would not only be valuable but could also enhance the reliability of obesity risk assessment indicators.

In this study, we exclusively investigated the association between GNB3 SNP (rs5443) allele distribution and two metrics: BMI and body fat percentage. However, obesity-related and NCDs-related characteristics extend beyond these metrics. Research on obesity-related genes typically examines SNP correlations with multiple parameters, including waist circumference, hip circumference, fasting glucose, blood pressure, total cholesterol and triglyceride levels [8,26,27]. A 2013 study on the Taiwanese population revealed that individuals with normal BMI and the GNB3 C/C genotype had higher average triglyceride and total cholesterol values. These findings align with our research which suggests that individuals with the GNB3 C/C genotype have a higher probability of having a BMI ≥ 27 and, in females, a body fat percentage ≥30 %. Future research should therefore expand upon this work by recruiting more participants and incorporating additional clinical and metabolic characteristics to further elucidate the role of the GNB3 C/C genotype in the Taiwanese population.

## 5. Conclusions

Modern society has witnessed a rapid increase in obesity, which is a major risk factor for numerous chronic diseases. Early genetic testing for obesity-related genes may enable individuals to proactively modify their lifestyles to prevent obesity and reduce the risk of NCDs. In our study, we demonstrated that Taiwanese individuals carrying the C/C homozygous genotype at nucleotide position 825 in exon 10 of the GNB3 gene are more likely to have elevated BMI and body fat percentage. Hence, we identified a potential genetic marker that could be incorporated into clinical health screenings to assess future obesity risk.

## Figures and Tables

**Table 1:** Genotype distribution and allele frequencies of GNB3 among 372 subjects by sex and age.

Age and gender		No. (%) of subjects				*P value*	No. (%) of subjects				*P value*
	
		Genotype					Genotype				
	
		C/C		C/T			C/C		T/T		
Sex	F	43	(23.8)	138	(76.2)	0.105	43	(36.1)	76	(63.9)	0.290
	M	27	(34.1)	54	(66.7)		27	(44.3)	34	(55.7)	
Age	<40	49	(26.3)	137	(73.7)	0.831	49	(40.2)	73	(59.8)	0.611
	≥40	21	(27.6)	55	(72.4)		21	(36.2)	37	(63.8)	

**Table 2:** Genotype distribution and allele frequencies of GNB3 among 372 subjects in different body mass index (BMI) subjects.

BMI (Kg/m^2^)	No. (%) of subjects				*P value*	No. (%) of subjects				*P value*
	
	Genotype					Genotype				
	
	C/C		C/T			C/C		T/T		
Normal	35	(22.9)	118	(77.1)	0.177	36	(31.3)	77	(68.7)	0.020
Overweight	17	(36.2)	30	(63.8)		17	(47.2)	19	(52.8)	
Obesity	18	(29.0)	44	(71.0)		18	(56.3)	14	(43.8)	
